# Perception of Healthcare Professionals towards Electronic-Prescribing at University of Gondar Comprehensive Specialized Hospital, Northwest Ethiopia: A Cross-Sectional Study

**DOI:** 10.1155/2024/6553470

**Published:** 2024-04-09

**Authors:** Faisel Dula Sema, Abel Getu Kebede, Girum Zeleke Soworsu, Tigist Tsegaye Mengistu, Hussien Endris Assen, Esileman Abdela Muche, Rahel Belete Abebe, Leila Kenzu Kemal, Abdisa Gemedi Jara, Abdulwase Mohammed Seid

**Affiliations:** ^1^Department of Clinical Pharmacy, School of Pharmacy, College of Medicine and Health Sciences, University of Gondar, Gondar, Ethiopia; ^2^Schools of Pharmacy, College of Medicine and Health Sciences, University of Gondar, Gondar, Ethiopia; ^3^Department of Anesthesia, School of Medicine, College of Medicine and Health Sciences, University of Gondar, Gondar, Ethiopia

## Abstract

**Background:**

Electronic-prescribing (e-prescribing) is the most recent technological advancement in the medication use process. Its adoption and consequent realization of its potential benefits, however, mainly depend on the healthcare professionals' perception, willingness to accept, and engagement with the technology.

**Objectives:**

This study is aimed at assessing the perception of healthcare professionals towards e-prescribing at the University of Gondar Comprehensive Specialized Hospital, Northwest Ethiopia, from June 1 to August 30, 2021.

**Method:**

A cross-sectional study was conducted using a simple random sampling technique. A self-administered questionnaire was used for data collection. Data were entered into and analyzed by using the Statistical Package for the Social Sciences (SPSS® (IBM Corporation)) version 24. Both descriptive and inferential statistics like the Kruskal-Wallis and Mann–Whitney tests were used for data analysis. A statistical significance was declared at a *p* value < 0.05.

**Result:**

From 401 participants, the majority of study participants had a neutral perception of e-prescribing. More than two-thirds (68.8%) of them had a neutral perception towards the perceived usefulness of e-prescribing with a median (interquartile range (IQR)) perceived usefulness of 43.0 (7.0) (maximum score = 60). The perceived ease of use of e-prescribing was also neutral in the case of more than three-fourths (79.8%) of participants with a median (IQR) perceived ease of use of 49.0 (6) (maximum score = 75). Similarly, more than half (56.6%) of the participants had a neutral perception towards the perceived fitness of e-prescribing with a median (IQR) perceived fitness of 15.0 (2.5) (maximum score = 15). The perception of the participants showed a significant difference based on their qualifications and work and computer use experience. Participants who heard about e-prescribing and e-prescribing software had a significantly higher mean rank score of perceived usefulness, perceived ease of use, and perceived fitness of e-prescribing. Participants who previously used e-prescribing had also a significantly higher mean rank score of perceived usefulness. *Conclusion and Recommendation*. The majority of healthcare professionals had a neutral perception of e-prescribing. The perception of healthcare professionals differs based on their qualifications, work and computer use experience, and their exposure to e-prescribing. The hospital should take all expectations and concerns of all HCPs into consideration and provide experience-sharing opportunities for all healthcare professionals who may potentially be involved in e-prescribing.

## 1. Introduction

Over many years, handwritten prescription has been a preferred communication method for physicians for transmitting decisions relating to medication therapy to pharmacists. In the last decade, however, electronic-prescribing (e-prescribing) has been taking hold as the most recent technological advancement over paper-based prescribing to generate, to transmit, and to fill prescription or prescription-related information between stakeholders either directly or through an intermediary including an e-prescribing network using electronic media or software [1–7]. The e-prescribing system provides prescribing drugs electronically that can be a stand-alone system or be integrated with the Electronic Health Record (EHR) system [6].

The potential benefits of e-prescribing are meant to extend to prescribers, payers, pharmacists, and patients [4]. Along with other health information technologies (HITs) such as HER and health information exchanges, the implementation of this application can end many problems of the paper-based prescribing process in terms of reducing prescribing errors, increasing efficiency, and healthcare cost savings [2, 4, 6, 8, 9]. Medication errors could be reduced to as little as a seventh of their previous level. Moreover, an estimated cost between $140 billion and $240 billion would be saved due to improved patient health outcomes and decreased patient visits over 10 years of practice [9].

E-prescribing is one part of the larger move to increased utilization of HITs [4]. It has been shown that most user groups perceive that e-prescribing would be facilitated by design and technical concerns, interoperability, content appropriate for the users, productivity, available resources, and attitude towards e-prescribing. However, “the digitalization process often is neither smooth nor successful” [3]; the lack of provider support, patient privacy, system errors, legal issues, cost related to its adaptation by health facilities, and related health workers have been significant barriers that often affect the success of its implementation [4, 6, 9–12]. It has also been shown that the same factor can be seen as a barrier or a facilitator depending on the project's own circumstances [3]. Moreover, the consequent realization of its benefits mainly depends on the potential end-user perception or attitude, willingness to accept, and engagement with the technology [13]. Similarly, their previous experience with paper-based prescription and computer use can greatly influence their attitude towards e-prescribing [11, 14].

Studies in developed prescribing [13, 15] and developing prescribing [16–18] countries, including Ethiopia [19], have focused on assessing the attitude of physicians towards e-prescribing [13, 15–18], who are the main prescribers in Ethiopia. Despite the perception of all involved parties that may be essential for the successful adoption of e-prescribing, there is scarce study report that includes the perspective of healthcare professionals (HCPs) other than physicians in Ethiopia. Moreover, whether or not there is a difference between the perceptions of different health professionals has not been merely reported. So this study is aimed at assessing the perceptions of HCPs towards e-prescribing at the University of Gondar Comprehensive Specialized Hospital (UoGCSH). Since the hospital has been in the implementation process of the electronic medical record, this study may provide immense contextual information for the hospital and all interested stakeholders.

## 2. Method

### 2.1. Study Design, Period, and Area

This cross-sectional study was conducted at UoGCSH from June 1 to August 30, 2021. The hospital is located 750 km northwest of Addis Ababa in the central Gondar administrative zone, Amhara National Regional State, Northwest Ethiopia. It was founded as Gondar Public Health College and Training Center with the involvement of the USAID, the WHO, and the Ministry of Public Health in 1954. Currently, the UoGCSH serves more than 13 million people in the catchment area [20]. Currently, the hospital is in the implementation phase of electronic medical records.

### 2.2. Population and Eligibility Criteria

HCPs working in the UoGCSH were the source population, whereas HCPs who were working in the hospital from June 1 to August 30, 2021, were the study population. HCPs involved in the prescribing process in the study period were included. However, HCPs who did not give consent were excluded.

### 2.3. Sample Size Determination and Sampling Procedure

The sample size was determined by using a single population proportion formula. *n* = (*Z*_*α*/2_)2 *P* (1 − *P*)/*W*2, where *Z*_*α*/2_ = 1.96, *P* = 50% (0.05), *W* = 0.05 (margin of error), and *n* is the number of prescribers to be sampled (sample size). *n* = (1.96)2(0.5) (1 − 0.5)/(0.05)2, where *n* = 384.16. *n* = *n* + 10%nonresponse = 384.16 + 10% × 384.16 = 384.16 + 38.416 = 423. In UoGCSH, the number of professionals who were involved in prescribing-related activities during the study period was 1174. A stratified simple random sampling technique was used to select the participants from their workplaces (supplementary file 1).

### 2.4. Study Variables

The dependent variable was the perception of HCPs towards the usefulness, ease of use, and fitness of e-prescribing. The independent variables were sociodemographic variables (age and sex), profession, year of working and computer use experience, and hearing about e-prescribing and e-prescribing software.

### 2.5. Definitions of Terms

A *prescription* is a written order by the doctor to the pharmacist. It has the status of a legal document [11].

*E-prescribing* is clinicians' computerized ordering of specific medication regimens for individual patients [21].

*HCPs* in this study are all healthcare workers who are involved in a prescription writing process including physicians, nurses, psychiatric nurses, anesthetists, optometrists, health officers, and physiotherapists excluding internship students and pharmacists.

### 2.6. Data Collection Procedure and Quality Control

Three pharmacists collected the data using a self-administered questionnaire adopted from previous studies [11, 14, 22]. The questionnaire included eight sections including background information, current prescribing activities, computer use personal experience, information about e-prescribing and e-prescribing software, perceived usefulness, perceived ease of use, perceived fitness, and exposure to e-prescribing. The response for sections two, three, five, six, and seven was measured on a Likert scale from 1 (strongly disagree) to 5 (strongly agree). The responses for the perceived usefulness, perceived ease of use, and perceived fitness of e-prescribing were categorized according to Bloom's cutoff point to negative, neutral, and positive [23]. Twelve [12] questions were concerning the perceived usefulness of e-prescribing with a possible total score of 12 to 60 (<36 as negative, 36-47 as neutral, and ≥48 as positive perception); 15 questions were about perceived ease of use of e-prescribing with a possible total score from 15 to 75 (<45 as negative, 45-59 as neutral, and ≥60 as positive perception), and 5 questions were about the perceived fitness of e-prescribing with a possible total score of 5 to 15 (<9 as negative, 9-11 as neutral, and ≥12 as positive). The questionnaire was pretested on 5% (21 individuals) of the sample size before data collection was started, and then, some adjustments were made to the qualification of the participants. The internal consistency of the instrument was assessed for reliability using Cronbach's coefficient alpha (≥0.7). Data was not collected on participants who were involved in the pretest, and the data obtained for the pretest was not included in the final analysis. The data was supervised on a daily base. One day of training was provided for the data collectors on the objective of the study, the contents of the questionnaire, and possible ethical considerations.

### 2.7. Data Processing and Analysis

After checking the completeness and consistencies, the data were entered, processed, and analyzed by using the Statistical Package for the Social Sciences (SPSS® (IBM Corporation)) version 24. Descriptive statistics like frequency, proportion, and median with interquartile range (IQR) were used. The normality of the data was tested using the Kolmogorov-Smirnov test and a skewness test. The inferential statistics were done by using the Mann–Whitney and Kruskal-Wallis tests. Comparisons of the KAP of the participants for each KAP question were done based on their sex, age, qualification, years of work experience, years of computer use experience, whether or not they heard about e-prescriptions and e-prescription software, and previous use of e-prescription (supplementary file 2). The comparison of the perception of participants was made by a Kruskal-Wallis test for groups having more than two categories and a Mann–Whitney *U* test for groups with two categories. Since the distributions of the participants' perception scores were not normally distributed across the different sociodemographic characteristics of the participants, the results of the Mann–Whitney *U* test and Kruskal-Wallis test were interpreted as mean rank score, and a statistical significance was declared at a *p* value < 0.05.

## 3. Result

### 3.1. Sociodemographic Characteristics of Study Participants

From 423 distributed questionnaires, 401 participants responded with a response rate of 94.8%. The majority of participants were male (63.8%) with a median (interquartile range) age of 29 (60) years. Around half (501.8) of the participants were physicians. Around half (48.4%) of the participants had 1-5 years of work experience (Table 1).

### 3.2. Current Prescribing Activities

About three-fourths (72.6%) of participants claimed that they were working with a high load of patients. Even though more than three-fourths (83.1%) of the participants claimed that their prescriptions are legible and more than half (56.7%) of them liked paper prescriptions, only less than half (46.2%) of them were able to track the continuity of their prescriptions. More than one-third (39.2%) of the participants disagreed that pharmacies incorrectly fill their prescriptions, and half (50.3%) of them responded that patients reported lost prescriptions requesting a replacement (Table 2).

### 3.3. E-Prescribing and Computer Use Experience

Only less than one-tenth (8.2%) of the participants used e-prescribing. However, more than half (57.6%) and one-third (35.7%) of the participants heard about e-prescribing and e-prescribing software, respectively. Moreover, around half (50.4%) of the participants had 1-5 years of computer use experience (Table 3). More than three-fourths (78.6%) and two-thirds (69%) of participants reported that they were comfortable with the use of computers and had a self-assessed good knowledge of computer use, respectively. Most of the participants (81.8%) use computers/laptops for professional and personal purposes. More than two-thirds (70.1%) of the participants regularly use computers at home; however, only about one-third (36.4%) of them use computers at the hospital (Table 4).

### 3.4. Perceived Usefulness, Ease of Use, and Fitness of E-Prescribing

**Figure 1:**
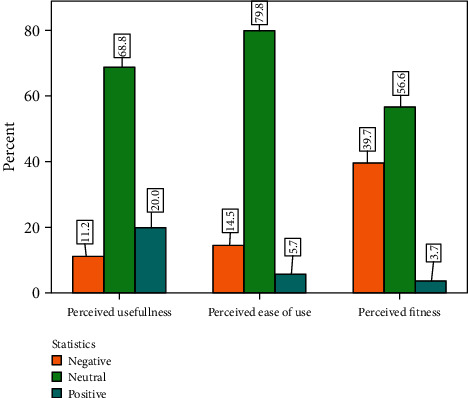
The perception of HCPs towards e-prescribing at UoGCSH from June 1 to August 30, 2021.

The median (interquartile range (IQR)) perception of the participants towards the usefulness of e-prescribing was 43.0 (7.0) from a possible total score from 12 to 60. The median (IQR) perception of the participants towards the ease of use of e-prescribing was 49.0 (6) from a possible total score of 15 to 75. Similarly, the median (IQR) perception of the participants towards the fitness of e-prescribing was 15.0 (2.5) from a possible total score from 5 to 15. Generally, more than two-thirds (68.8%), three-fourths (79.8%), and half (56.6%) of the participants had a neutral perception towards the perceived usefulness, ease of use, and fitness of e-prescribing, respectively (Figure 1).

The majority of the study participants thought that the ability to send e-prescribing would be good (66.3%) and lead to safer prescribing (73.6%). A vast majority of participants also liked getting notified when there is a potential chance of drug-drug interactions (62.8%), whether the patients receive the prescribed medication from the pharmacies (60.1%), and what other doctors prescribe for coexisting illnesses (61.4%). Around two-thirds of the participants agreed that the storage of personal healthcare information in a database could be used for research purposes (66.8%) and that e-prescribing could decrease the costs for the healthcare system (65.3%) (Table 5).

About two-thirds of the participants agreed that using e-prescribing means easier prescribing (66.8%), and it is fast and will save time (67.9%). Around a quarter (23.9%) of the participants felt that it would affect their workflow, and around half (48.4%) of them considered that it would cause technical problems and require regular technical assistance. The majority of the participants thought that it would be easier to renew prescriptions electronically (59.6%) and that e-prescribing would help in detecting medication misuse and diversion (62.8%). However, around half (50.2%) of them claimed that they would like to meet the patients in person rather than give automatic refills. Moreover, more than two-thirds (70.3%) of them considered that the pharmacies need to be well equipped to fit into the e-prescribing network (Table 5).

Even though more than three-fourths (78.6%) of the participants agreed to accept e-prescribing once it is adopted in the institution, one-third (33.9%) of them felt that it would not be ideal for a high-volume center. According to more than two-thirds (72.6%) and more than three-fourths (79.3%) of participants, the facilities in the institution need rapid modifications and there need to be orientation classes and mass training programs before the adoption of e-prescribing in the institution, respectively (Table 5).

### 3.5. Comparison of the Perceived Usefulness, Ease, and Fitness of E-Prescribing Based on Different Characteristics of the Study Participants

The perception of the participants towards the usefulness of e-prescribing showed a significant difference based on their qualifications (*p* value = 0.007), years of work experience (*p* value = 0.034), and years of computer use experience (*p* value = 0.004). Similarly, their perception of the ease of use of e-prescribing showed a significant difference based on the work experience of the participants (*p* value = 0.042). The perception of the participants towards the fitness of e-prescribing also showed a significant difference based on their work experience (*p* value = 0.003).

The mean rank score of participants' perception of the usefulness of e-prescribing was significantly higher among those who heard about e-prescribing (*p* value < 0.001), e-prescribing software (*p* value < 0.001), and previous use of e-prescribing (*p* value = 0.001). The mean rank score of participants' perception of the ease of e-prescribing was significantly higher among those who heard about e-prescribing (*p* value = 0.002) and e-prescribing software (*p* value = 0.001). The mean rank score of participants' perception of the fitness of e-prescribing was also significantly higher among those who heard about e-prescribing (*p* value = 0.002) and e-prescribing software (*p* value = 0.001) (Table 6). The table which contains both significant and no significant association is provided as a supplementary file (supplementary file 2).

## 4. Discussion

This study assessed the perception of HCPs towards e-prescribing in three domains, perceived usefulness, perceived ease of use, and perceived fitness of the e-prescribing system. Generally, the majority of participants had a neutral perception of the perceived usefulness, ease of use, and fitness of e-prescribing. However, previous studies in Ethiopia [24], Pakistan [25], Jordan [26], and the USA [14] reported that a majority of participants had a positive attitude towards e-prescribing. The difference may be due to variations in the study population, study settings, and stage of e-prescribing system implementation. So the institution should work on strategies that can improve the perception of the HCPs who may potentially be involved in e-prescribing. It is encouraging that the perception of HCPs may be improved after implementation [14]. However, when implementing the e-prescribing system, its benefits, barriers, and adopting factors that can affect the success of the implementation need to be considered [6].

In this study, the majority of participants responded that they would accept it if it is adopted in the institution. This finding is in line with the study conducted in Turkey [18], Jordan [26], and Ireland [27]. The majority of them expected that having the capability to send e-prescribing is good, using e-prescribing would be safer and save time than paper-based prescription, the e-prescribing data can be used for research purposes, using e-prescribing decreases costs of the healthcare, and e-prescribing means better service to the patients. Similar trends have also been reported in many previous studies [11, 15, 28, 29]. The majority of the study participants also would like to get alerted about drug-drug interaction, know that the patient received the medication from the pharmacy, and what other doctors are prescribing to their patient. In addition, the majority reported that their work would be easier if they used e-prescribing, and it would be easy to renew prescriptions and identify diversion and misuse of medicines with e-prescribing. For the better acceptance and widespread adoption of the e-prescribing system, e-prescribing should be designed as user-friendly and to the performance expectation of HCPs in the form of improved productivity and a more effective prescribing process [13, 16].

Study participants, however, also had many concerns about the adoption of the e-prescribing system. It is supported by many published reports [9, 17, 21, 30, 31]. The storage of more and more personal healthcare information and its availability in databases, data abuse, a sense of being controlled, and security issues were considered a problem. Some HCPs disliked the fact that patients were not getting the prescription in their hand and automatically filling prescriptions. Fear of disruption in workflow, time wastage due to technical problems, difficulty to changing/canceling e-prescribing, the complicity of prescribing through software, and thinking of e-prescribing as the not ideal system for a center with a high patient load may create some additional challenges to implementation. So adopting e-prescribing should seriously consider the concerns of all HCPs. For the successful implementation of the e-prescribing system, regular technical assistance, equipping the pharmacies with medications, and a drastic modification in the facilities of the institution are required [8]. Adopting a more user-friendly e-prescribing system may require reforming work processes, which in turn would enhance the effectiveness of the HCPs' prescription process [13]. The iterative rollout may enable the HCPs to overcome the initial anxiety associated with adoption [14]. Moreover, there need to be orientation classes, mass training programs, and experience-sharing opportunities for all involved parties [8, 14, 31].

The perception of the participants towards the usefulness of e-prescribing showed a significant difference based on computer use experience. It is supported by the study conducted in Kerala, India [11]. In addition, the perceived usefulness of e-prescribing significantly varied based on the qualification of the participants. It is consistent with the findings reported by the study conducted in Pakistan [25]. The mean rank score of participants' perception of the ease of use and fitness of e-prescribing was significantly higher among those who heard about e-prescribing. This is consistent with the study conducted in Kerala, India [11].

## 5. Limitations of the Study

Despite this study trying to consider a wider range of HCPs which may make it relatively unique, being a single-center study may limit its generalizability. Moreover, including the perspective of pharmacists and patients would have been great.

## 6. Conclusion and Recommendation

In this study, the majority of HCPs had a neutral perception of the perceived usefulness, ease of use, and fitness of e-prescribing. The perception of HCPs towards the perceived usefulness, ease of use, and fitness showed significant differences based on their qualification and work and computer use experience. HCPs who heard about e-prescribing, e-prescribing software, and previous use of e-prescribing had a better perception of the perceived usefulness, ease of use, and fitness of e-prescribing.

The hospital should take all expectations and concerns of all HCPs into consideration for the successful adoption of the e-prescribing system. The hospital could provide training before the adoption of e-prescribing in the institution. The hospital should also create an opportunity for experience sharing with all HCPs who are potentially involved in e-prescribing in the institution to increase their exposure to the e-prescribing system and e-prescribing software. Future researchers could focus on the perspectives of pharmacists and patients.

## Figures and Tables

**Table 1:** Sociodemographic characteristics of HCPs at UoGCSH, Northwest Ethiopia (*N* = 401).

Characteristics	Categories	Frequency (%)
Sex	Male	256 (63.8)
	Female	145 (36.2)

Age group in year	≤29	228 (56.9)
	30-39	156 (38.9)
	>40	17 (4.2)

Qualification	Physicians	208 (51.8)
	Nurse	120 (29.9)
	Psychiatric nurse	15 (3.7)
	Anesthetist	7 (1.7)
	Optometrist	31 (7.7)
	Health officer	3 (0.7)
	Physiotherapist	17 (4.2)

Years of work experience	<1 year	75 (18.7)
	1-5 years	194 (48.4)
	6-10 years	104 (25.9)
	10-15 years	21 (5.2)
	16-20	4 (1.0)
	>20	3 (0.7)

HCPs: healthcare professionals; UoGCSH: University of Gondar Comprehensive Specialized Hospital; *N*: frequency.

**Table 2:** Current prescribing activities of HCPs at UoGCSH, Northwest Ethiopia (*N* = 401).

Variables	Disagree, *N* (%)	Neutral, *N* (%)	Agree, *N* (%)
The patient load for me is high	42 (12.5)	68 (17)	291 (72.6)
The prescription written by me is clear	27 (6.7)	41 (10.2)	333 (83.1)
I like paper prescription	59 (14.7)	115 (28.7)	227 (56.6)
Pharmacies clear any doubt in my prescription	80 (20)	123 (30.7)	198 (49.4)
Usually able to track the continuity of my prescriptions	83 (20.7)	114 (28.4)	204 (50.9.2)
The prescription written by me is altered sometimes	218 (54.3)	85 (21.2)	98 (24.5)
Prescription pads were stolen sometimes	217 (54.1)	94 (23.4)	90 (22.4)
Pharmacies incorrectly fill my prescriptions sometimes	161 (39.2)	109 (27.2)	131 (32.7)
Patients reported lost prescriptions requesting a replacement	97 (24.2)	102 (25.4)	202 (50.3)

HCPs: healthcare professionals; UoGCSH: University of Gondar Comprehensive Specialized Hospital; *N*: frequency.

**Table 3:** Computer use and e-prescribing experience of HCPs at UoGCSH, Northwest Ethiopia (*N* = 401).

Variables	Category	Frequency (%)
Year of computer use experience	<1 year	82 (20.4)
	1-5 years	202 (50.4)
	6-10 years	86 (21.4)
	10-15 years	14 (3.5)
	>15 years	17 (4.2)

Heard about e-prescribing	Yes	231 (57.6)
	No	170 (42.4)

Heard about e-prescribing software	Yes	143 (35.7)
	No	258 (64.3)

Previous use of e-prescribing	Yes	33 (8.2)
	No	368 (91.8)

HCPs: healthcare professionals; UoGCSH: University of Gondar Comprehensive Specialized Hospital; *N*: frequency; e-prescribing: electronic-prescribing.

**Table 4:** Computer use activities of HCPs at UoGCSH, Northwest Ethiopia (*N* = 401).

Variables	Disagree, *N* (%)	Neutral, *N* (%)	Agree, *N* (%)
Comfortable with the use of computers	35 (8.7)	51 (12.7)	315 (78.6)
Computers use for professional and personal purposes	31 (7.7)	42 (10.5)	328 (81.8)
Use computers in the home	55 (13.7)	65 (16.2)	281 (70.1)
Use computers at the hospital	160 (39.9)	95 (23.7)	146 (36.4)
Good knowledge regarding the use of computers	41 (41.7)	83 (20.7)	277 (69)

HCPs: healthcare professionals; UoGCSH: University of Gondar Comprehensive Specialized Hospital; *N*: frequency.

**Table 5:** Perceived usefulness, ease, and fitness of e-prescribing among HCPs at UoGCSH, Northwest Ethiopia (*N* = 401).

Variables	Disagree, *N* (%)	Neutral, *N* (%)	Agree, *N* (%)
Perceived usefulness of e-prescribing			
Having the capability to send e-prescribing is good	53 (13.2)	82 (20.4)	266 (66.3)
Compared to paper prescriptions, e-prescribing will save time	38 (9.5)	101 (25.2)	262 (65.3)
Compared to paper prescriptions, e-prescribing will be safer	29 (7.2)	77 (19.2)	295 (73.6)
Compared to paper prescription, e-prescribing means better service to the patients	38 (9.5)	99 (24.7)	264 (65.8)
I like getting alerted about drug-drug interaction	37 (9.2)	112 (27.9)	252 (62.8)
E-prescribing will enable me to know that the patient has received the medication from the pharmacy	38 (9.5)	122 (30.4)	241 (60.1)
E-prescribing will enable me to see what other doctors are prescribing to my patient which I would like to know	47 (11.7)	108 (26.9)	246 (61.4)
I am worried that my work will be controlled when sending e-prescribing	140 (34.9)	154 (38.4)	107 (26.6)
It is a problem that more and more personal healthcare information is stored and available in databases	111 (27.7)	126 (31.4)	164 (40.9)
I am worried about data abusing	103 (25.7)	148 (36.9)	150 (37.4)
It is good that more and more data is available so that we can carry out health-related research	42 (10.5)	91 (22.7)	268 (66.8)
E-prescribing reduces costs for the health system	43 (10.7)	96 (23.9)	262 (65.3)

Perceived ease of use of e-prescribing			
My work will be easier if I use e-prescribing	48 (12)	85 (21.2)	268 (66.8)
E-prescribing is fast and will save time	45 (11.2)	84 (20.9)	272 (67.9)
E-prescribing is fast but might cause a lot of time wastage due to technical problems	200 (49.9)	122 (30.4)	79 (19.7)
Patients will be worried that I am referring Internet and prescribing	84 (20.9)	141 (35.2)	176 (43.9)
E-prescribing improves patient satisfaction	61 (15.2)	161 (40.1)	179 (44.7)
I do not like the fact that patients are not getting the prescription in their hand	137 (34.2)	138 (34.4)	126 (31.4)
E-prescribing will affect my workflow	165 (41.2)	140 (34.9)	96 (23.9
E-prescribing will require technical assistance regularly	99 (24.7)	108 (26.9)	194 (48.4)
It will be easy to renew prescriptions	47 (11.7)	115 (28.7)	239 (59.6)
I like to see the patients in person and assess them rather than automatically fill prescriptions	82 (20.4)	118 (29.4)	201 (50.2)
With e-prescribing, it is easy to identify the diversion and misuse of medicines	47 (11.7)	102 (25.4)	252 (62.8)
Prescribing through software is complicated	148 (36.9)	128 (31.9)	125 (31.2)
The pharmacies should be equally equipped with medications for the success of e-prescribing	36 (9)	83 (20.7)	282 (70.3)
It will be difficult to change/cancel e-prescribing	208 (51.9)	123 (30.7)	70 (17.4)
Someone might log in to my ID and send unauthorized prescriptions	148 (36.9)	128 (31.9)	125 (31.2)

Perceived fitness of e-prescribing			
I will accept e-prescribing if it is adopted in the institution	28 (7)	18 (14.5)	315 (78.6)
I think the facilities in the institution will need drastic modifications	35 (8.8)	75 (18.8)	290 (72.4)
I do not think e-prescribing is ideal for a center with a high patient load	166 (41.4)	99 (24.7)	136 (33.9)
There need to be orientation classes and mass training before the adoption of e-prescribing in the institution	32 (8)	51 (12.7)	318 (79.3)

HCPs: healthcare professionals; UoGCSH: University of Gondar Comprehensive Specialized Hospital; *N*: frequency; e-prescribing: electronic-prescribing.

**Table 6:** Comparison of the perception of HCPs based on their characteristics (*N* = 401).

Variables	Category	Frequency (%)	Attitude (mean rank score)	Mann–Whitney/Kruskal-Wallis test	*p* value	*Z*-score
*Perceived usefulness*						
Qualification	Physicians	208	211.75	17.671	0.007	
	Nurse	120	174.86			
	Psychiatric nurse	15	218.40			
	Anesthetist	7	164.93			
	Optometrist	31	231.87			
	Health officer	3	354.67			
	Physiotherapist	17	170.03			
Years of work experience	<1 year	75	175.91	12.080	0.034	
	1-5 years	194	207.82			
	6-10 years	104	194.09			
	10-15 years	21	224.29			
	16-20	4	307.63			
	>20	3	321.67			
Year of computer use experience	<1 year	82	186.82	15.495	0.004	
	1-5 years	202	186.51			
	6-10 years	86	232.34			
	10-15 years	14	238.89			
	>15 years	17	251.88			
Heard about e-prescriptions	Yes	231	235.02	11776.00	<0.001	-6.863
	No	170	154.77			
Heard about e-prescription software	Yes	143	246.91	11882.50	<0.001	-5.914
	No	258	175.56			
Previous use of e-prescription	Yes	33	264.92	3962.50	0.001	-3.313
	No	368	195.27			

*Perceived ease of use*						
Years of work experience	<1 year	75	170.88	11.516	0.042	
	1-5 years	194	208.46			
	6-10 years	104	205.03			
	10-15 years	21	237.17			
	16-20	4	95.13			
	>20	3	219.83			
Heard about e-prescriptions	Yes	231	216.02	16165.00	0.002	-3.033
	No	170	180.59			
Heard about e-prescription software	Yes	143	225.82	14897.50	0.001	-3.201
	No	258	187.24			

*Perceived fitness*						
Years of work experience	<1 year	75	170.88	11.516	0.003	
	1-5 years	194	208.46			
	6-10 years	104	205.03			
	10-15 years	21	237.17			
	16-20	4	95.13			
	>20	3	219.83			
Heard about e-prescriptions	Yes	231	216.46	16064.00	0.002	-3.140
	No	170	179.99			
Heard about e-prescription software	Yes	143	227.17	14705.00	0.001	-3.395
	No	143	227.17			

HCPs: healthcare professionals; *N*: frequency; e-prescribing: electronic-prescribing.

## Data Availability

The authors confirm that the data used to support the finding of this study will be available from the corresponding author upon request.

## Supplementary Materials

Supplementary 1 Sampling procedure employed among HCPs at UoGCSH, Northwest Ethiopia from June 1 to August 30, 2021.

Supplementary 2 Comparison of the perception of HCPs based on their characteristics.
