# Supplementary figures and images for: Perception of Healthcare Professionals towards Electronic-Prescribing at University of Gondar Comprehensive Specialized Hospital, Northwest Ethiopia: A Cross-Sectional Study
Source: Biomed Res Int. 2024 Apr 9;2024:6553470. doi: 10.1155/2024/6553470 (PMC11022515; doi:10.1155/2024/6553470)

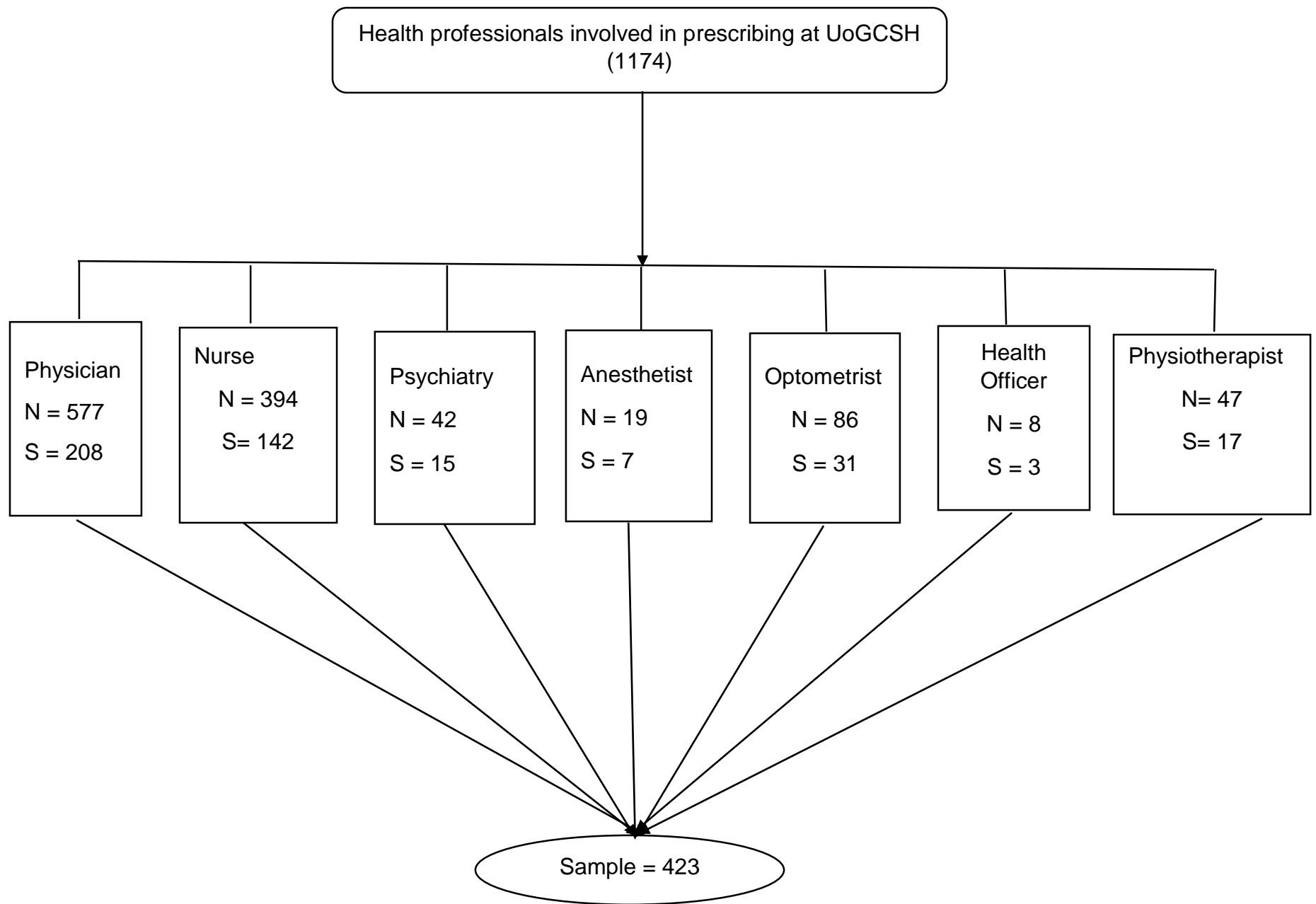

Supplement: Supplementary 1 — Sampling procedure employed among HCPs at UoGCSH, Northwest Ethiopia from June 1 to August 30, 2021. [file 6553470.f1.pdf]
