# Supplementary material for: Perception of Healthcare Professionals towards Electronic-Prescribing at University of Gondar Comprehensive Specialized Hospital, Northwest Ethiopia: A Cross-Sectional Study
Source: Biomed Res Int. 2024 Apr 9;2024:6553470. doi: 10.1155/2024/6553470 (PMC11022515; doi:10.1155/2024/6553470)
Supplement: Supplementary 2 — Comparison of the perception of HCPs based on their characteristics. [file 6553470.f2.docx]

Supplementary file 2: Comparison of the perception of HCPs based on their characteristics. (N = 401)

| Variables | Category | Frequency (%) | Attitude (mean rank score) | Mann.Whitney /Kurkulias test | P-value | Z-score |
| --- | --- | --- | --- | --- | --- | --- |
| Perceived usefulness | | | | | | |
| Sex | Male | 256 | 206.65 | 16857.50 | 0.155 | -1.421 |
|  | Female | 145 | 189.57 |  |  |  |
| Age group in year | ≤ 29 | 229 | 200.26 | 19524.50 | 0.883 | -0.148 |
|  | >30 | 172 | 201.99 |  |  |  |
| Qualification | Physicians | 208 | 211.75 | 17.671 | 0.007 |  |
|  | Nurse | 120 | 174.86 |  |  |  |
|  | Psychiatry Nursing | 15 | 218.40 |  |  |  |
|  | Anesthetist | 7 | 164.93 |  |  |  |
|  | Optometrist | 31 | 231.87 |  |  |  |
|  | Health Officer | 3 | 354.67 |  |  |  |
|  | Physiotherapist | 17 | 170.03 |  |  |  |
|  | Nurse | 120 | 174.86 |  |  |  |
|  | Psychiatry Nursing | 15 | 218.40 |  |  |  |
| Years of work experience | <1 year | 75 | 175.91 | 12.080 | 0.034 |  |
|  | 1-5 years | 194 | 207.82 |  |  |  |
|  | 6-10 years | 104 | 194.09 |  |  |  |
|  | 10 -15 years | 21 | 224.29 |  |  |  |
|  | 16-20 | 4 | 307.63 |  |  |  |
|  | >20 | 3 | 321.67 |  |  |  |
| Year of computer use experience | <1 year | 82 | 186.82 | 15.495 | 0.004 |  |
|  | 1-5 years | 202 | 186.51 |  |  |  |
|  | 6-10 years | 86 | 232.34 |  |  |  |
|  | 10-15 years | 14 | 238.89 |  |  |  |
|  | >15 years | 17 | 251.88 |  |  |  |
| Heard about e-Prescriptions | Yes | 231 | 235.02 | 11776.00 | <0.001 | -6.863 |
|  | No | 170 | 154.77 |  |  |  |
| Heard about e-prescription software | Yes | 143 | 246.91 | 11882.50 | <0.001 | -5.914 |
|  | No | 258 | 175.56 |  |  |  |
| Previous use of e-prescription | Yes | 33 | 264.92 | 3962.50 | 0.001 | -3.313 |
|  | No | 368 | 195.27 |  |  |  |
| Perceived ease | | | | | | |
| Sex | Male | 256 | 199.26 | 18113.50 | 0.774 | -0.288 |
|  | Female | 145 | 202.71 |  |  |  |
| Age group in year | ≤ 29 | 229 | 204.22 | 18955.50 | 0.519 | -0.645 |
|  | >30 | 172 | 196.71 |  |  |  |
| Qualification | Specialist | 51 | 180.44 | 14.796 | 0.063 |  |
|  | Resident | 102 | 216.26 |  |  |  |
|  | General Practitioner | 55 | 170.80 |  |  |  |
|  | Nurse | 120 | 203.91 |  |  |  |
|  | Psychiatry Nursing | 15 | 212.90 |  |  |  |
|  | Anesthetist | 7 | 217.57 |  |  |  |
|  | Optometrist | 31 | 243.32 |  |  |  |
|  | Health Officer | 3 | 212.33 |  |  |  |
|  | Physiotherapist | 17 | 151.74 |  |  |  |
| Years of work experience | <1 year | 75 | 170.88 | 11.516 | 0.042 |  |
|  | 1-5 years | 194 | 208.46 |  |  |  |
|  | 6-10 years | 104 | 205.03 |  |  |  |
|  | 10 -15 years | 21 | 237.17 |  |  |  |
|  | 16-20 | 4 | 95.13 |  |  |  |
|  | >20 | 3 | 219.83 |  |  |  |
| Year of computer use experience | <1 year | 82 | 201.95 | 2.909 | 0.573 |  |
|  | 1-5 years | 202 | 197.27 |  |  |  |
|  | 6-10 years | 86 | 215.43 |  |  |  |
|  | 10-15 years | 14 | 165.32 |  |  |  |
|  | >15 years | 17 | 197.12 |  |  |  |
| Heard about e-Prescriptions | Yes | 231 | 216.02 | 16165.00 | 0.002 | -3.033 |
|  | No | 170 | 180.59 |  |  |  |
| Heard about e-prescription software | Yes | 143 | 225.82 | 14897.50 | 0.001 | -3.201 |
|  | No | 258 | 187.24 |  |  |  |
| Previous use of e-prescription | Yes | 33 | 197.29 | 5949.50 | 0.847 | -0.193 |
|  | No | 368 | 201.33 |  |  |  |
| Perceived fitness | | | | | | |
| Sex | Male | 256 | 204 | 17304.00 | 0.305 |  |
|  | Female | 144 | 192.67 |  |  |  |
| Age group in year | ≤ 29 | 229 | 196.73 | 18716.50 | 0.391 | -0.858 |
|  | >30 | 172 | 206.68 |  |  |  |
| Qualification | Specialist | 51 | 180.44 | 14.796 | 0.063 |  |
|  | Resident | 102 | 216.26 |  |  |  |
|  | General Practitioner | 55 | 170.80 |  |  |  |
|  | Nurse | 120 | 203.91 |  |  |  |
|  | Psychiatry Nursing | 15 | 212.90 |  |  |  |
|  | Anesthetist | 7 | 217.57 |  |  |  |
|  | Optometrist | 31 | 243.32 |  |  |  |
|  | Health Officer | 3 | 212.33 |  |  |  |
|  | Physiotherapist | 17 | 151.74 |  |  |  |
| Years of work experience | <1 year | 75 | 170.88 | 11.516 | 0.003 |  |
|  | 1-5 years | 194 | 208.46 |  |  |  |
|  | 6-10 years | 104 | 205.03 |  |  |  |
|  | 10 -15 years | 21 | 237.17 |  |  |  |
|  | 16-20 | 4 | 95.13 |  |  |  |
|  | >20 | 3 | 219.83 |  |  |  |
| Year of computer use experience | <1 year | 82 | 201.95 | 2.909 | 0.573 |  |
|  | 1-5 years | 202 | 197.27 |  |  |  |
|  | 6-10 years | 86 | 215.43 |  |  |  |
|  | 10-15 years | 14 | 165.32 |  |  |  |
|  | >15 years | 17 | 197.12 |  |  |  |
| Heard about e-Prescriptions | Yes | 231 | 216.46 | 16064.00 | 0.002 | -3.140 |
|  | No | 170 | 179.99 |  |  |  |
| Heard about e-prescription software | Yes | 143 | 227.17 | 14705.00 | 0.001 | -3.395 |
|  | No | 143 | 227.17 |  |  |  |
| Previous use of e-prescription | Yes | 33 | 238.36 | 4839.00 | 0.051 | -1.950 |
|  | No | 368 | 197.65 |  |  |  |
